# RETRACTION: Skin Boosters: Definitions and Varied Classifications

**DOI:** 10.1111/srt.70297

**Published:** 2025-11-21

**RETRACTION**: K.‐H. Yi, W. Winayanuwattikun, S.‐Y. Kim, J. Wan, V. Vachatimanont, A. I. Putri, I. J. Hidajat, Y. Yogya, and R. Pamela, “Skin Boosters: Definitions and Varied Classifications,” *Skin Research and Technology* 30, no. 3 (2024): e13627, https://doi.org/10.1111/srt.13627.

The above article, published online on 13 March 2024 in Wiley Online Library (wileyonlinelibrary.com), has been retracted by agreement between *Skin Research and Technology*; and John Wiley & Sons Ltd. The article was accepted for publication following an insufficient peer review process that does not meet the standards of the journal's peer review policy. Furthermore, the article contains several images sourced from the internet and/or previously published literature without appropriate citation or indication that permission for reuse has been obtained. Accordingly, the article has been retracted. The corresponding author, Yuri Yogya, disagreed with the decision of retraction; no confirmation was obtained by the remaining co‐authors.

